# Cell-cell fusion limits activation of the unfolded protein response induced by the Nipah virus glycoproteins

**DOI:** 10.1128/jvi.01046-25

**Published:** 2025-12-11

**Authors:** Paula Jordan, Sören Heyer, Julian Hüther, Ilka Fischer, Nico Becker, Andrea Maisner

**Affiliations:** 1 Institute of Virology, Philipps University Marburg 489120 , Marburg, Germany; University Medical Center Freiburg, Freiburg, Germany

**Keywords:** Nipah virus, glycoproteins, UPR, XBP1, syncytia, fusion

## Abstract

The productive replication of highly pathogenic Nipah virus (NiV) relies on the host cell for the synthesis and correct folding of the viral glycoproteins, which can cause ER stress and activation of the unfolded protein response (UPR). While the UPR can exert proviral functions by restoring ER homeostasis, it can also have antiviral effects. Here, we show that irreversible ER stress induced by thapsigargin resulted in broad expression of UPR target genes and potently inhibited NiV infection. The finding that UPR target gene upregulation was not detectable in NiV-infected cells at 18 h p.i., raised the question of how NiV regulates UPR activation to prevent thapsigargin-like antiviral effects. To address this, we analyzed the effects of NiV glycoprotein expression on UPR activation and found that both NiV glycoproteins F and G, like many other viral glycoproteins, activated the highly conserved IRE1/XBP1 branch of the UPR. Interestingly, upon coexpression of both NiV glycoproteins and thereby induced cell-cell fusion, the activation did not increase. Instead, UPR activation relatively decreased with increasing syncytium formation, an effect that was not observed if cell-cell fusion was blocked. These results support the idea that syncytium formation limits UPR activation despite ongoing viral glycoprotein synthesis. This as yet undescribed mechanism is likely a fusion-dependent countermeasure to prevent an overload of the ER folding capacity by dilution and suggests that NiV-induced syncytium formation not only is an important way to promote NiV spread from cell-to-cell but also regulates ER stress to limit potential UPR-induced antiviral responses.

**IMPORTANCE:**

The unfolded protein response (UPR) is a cellular signaling pathway to counteract ER stress. Many enveloped viruses, which force the infected cell to synthesize high amounts of viral surface glycoproteins, induce the UPR but regulate its activation by diverse strategies to prevent UPR-mediated antiviral effects. To date, nothing is known about UPR activation in infections with Nipah virus (NiV), a highly pathogenic member of the *Paramyxoviridae* family. Here, we demonstrate that NiV glycoproteins activate the IRE1/XBP1 branch of the UPR. However, this activation is limited by the cell-cell fusion mediated by the glycoproteins, probably due to dilution effects. This study is the first to investigate the interplay between NiV and UPR activation and proposes a novel strategy by which fusogenic viruses may limit the ER stress responses triggered by their glycoproteins.

## INTRODUCTION

Nipah virus (NiV) is a biosafety level 4 (BSL-4) classified henipavirus in the family *Paramyxoviridae* that causes highly fatal respiratory and encephalitic diseases (1). As a bat-derived emerging virus, it is listed on the WHO blueprint list of priority pathogens due to its potential to be transmitted from human-to-human after spillover events and to cause human diseases with high mortality rates (2–5).

NiV is an enveloped virus with a negative-sense, single-stranded RNA genome which encodes for six structural proteins. The nucleoprotein (N), the phosphoprotein (P), and the RNA-dependent RNA polymerase (L) encapsidate the viral RNA and form the helical nucleocapsid. The matrix protein (M) at the inner leaflet of the virus envelope interacts with the viral nucleocapsid and the two surface glycoproteins (6, 7). The NiV glycoprotein G, a type II transmembrane protein, is responsible for binding to the host cell receptors ephrin-B2/B3 (8–10), while the NiV fusion protein (F), a type I transmembrane glycoprotein, mediates fusion of the viral envelope with the host cell membrane during virus entry (11). In infected cells, the NiV surface glycoproteins are synthesized, folded, glycosylated, and oligomerized in the endoplasmic reticulum (ER). The newly synthesized NiV F and G proteins are then transported to the plasma membrane via the secretory pathway. The fusion-inactive precursor F_0_ is subsequently endocytosed to be proteolytically cleaved by cathepsin B/L at an acidic pH in endosomes to generate fusion-active F_1/2_, which is subsequently retransported to the plasma membrane (12, 13). Surface-expressed NiV glycoprotein complexes formed by fusogenic F_1/2_ and receptor-binding G proteins (F/G complexes) are incorporated into virus particles and induce pH-independent cell-cell fusion and syncytium formation. The formation of multinucleated cells allows a rapid spread of NiV from cell-to-cell and is a hallmark of NiV infection *in vitro* and *in vivo* (14, 15).

Viral infections often lead to the activation of cellular stress responses by overloading the cellular machinery for viral replication (16). One of the major stress inducers is the abundant *de novo* synthesis of viral glycoproteins in the ER, which exceeds the ER folding capacity. Since an accumulation of unfolded proteins in the ER leads to the recruitment of chaperones to enable their correct folding, the unfolded protein response (UPR) is activated (17, 18). The sensing of ER stress occurs by three transmembrane stress transducers, the inositol-requiring enzyme 1 α (IRE1), the activating transcription factor 6 (ATF6), and the protein kinase R (PKR)-like ER kinase (PERK), with IRE1 being the most conserved UPR signal transducer that is induced by numerous viral glycoproteins (16, 17, 19). Upon the recruitment of ER chaperones such as BiP by an excess of unfolded or misfolded proteins, IRE1 undergoes oligomerization and autophosphorylation. This enables its activity as an endoribonuclease that specifically cleaves the X-box binding protein 1 (XBP1) mRNA at two consensus sites, leading to the excision of a 26-nucleotide intron. The XBP1s (spliced isoform) mRNA is then translated into the transcription factor XBP1s, which translocates to the nucleus and can activate genes involved in ER-associated protein degradation (ERAD), ER chaperones and ER expansion. XBP1 is, thus, the key factor in alleviating ER stress by increasing the ER folding capacity. If the ER homeostasis cannot be reestablished, sustained UPR activation ultimately leads to apoptosis, which is primarily mediated by the PERK pathway of the UPR (17, 18, 20, 21). To avoid such an UPR-related premature cell death, most pathogenic viruses that induce the UPR via viral glycoprotein-induced ER stress have evolved strategies to prevent or modulate UPR activation to ensure productive replication, or even hijack the UPR as a proviral factor (22–25). Thus, ER stress regulation is often tightly linked to pathogenesis and virulence (16). As the specific regulatory interactions of highly pathogenic NiV and the host cell’s ER stress machinery are yet unknown, this study aimed to elucidate the interplay between NiV and the UPR.

## RESULTS

### The ER stress inducer thapsigargin blocks NiV infection

To first determine whether UPR activation generally has a positive or negative effect on NiV replication, we tested the effect of thapsigargin (Tg), a chemical UPR inducer that irreversibly interrupts the tightly regulated calcium homeostasis of the ER and activates all three UPR branches (26–28). For this purpose, Vero76 cells were infected with NiV at a multiplicity of infection (MOI) of 0.1. 1 h post infection (p.i.), the virus inoculum was removed and replaced by media containing 0.05% DMSO (control) or 500 nM Tg, the optimal nontoxic Tg concentration for UPR induction in Vero cells (29). At 24 h p.i., supernatants were collected for virus titration and the infected cells were fixed and stained to visualize syncytium formation. Additionally, cell lysates were collected for RNA isolation and western blot analyses.

**Fig 1:**
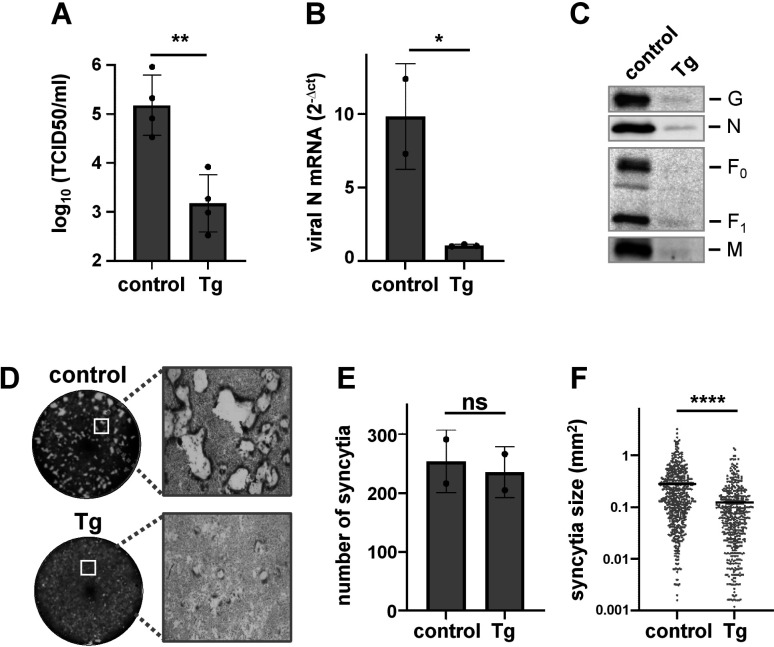
Thapsigargin inhibits NiV replication. Vero76 cells were infected with NiV at a multiplicity of infection (MOI) of 0.1. At 1 h p.i., medium containing 0.05% DMSO (control) or 500 nM thapsigargin (Tg) was added. (**A**) At 24 h p.i., infectious virus titers in the cell supernatants were determined using the TCID50 method. Virus titers are shown as mean ± standard deviation (SD). (**B**) To quantitate viral mRNA synthesis, total RNA was isolated from cell lysates and reverse transcribed using oligo(dT) primers. cDNA was then analyzed by quantitative real-time PCR using specific primers for NiV N. Ct values were normalized to the internal control (tubulin) and are shown as 2^−Δct^. Error bars indicate SD. (**C**) Cell lysates were collected 24 h p.i. and subjected to western blot analysis. NiV N, F, G, and M were stained with respective specific antibodies, biotinylated secondary antibodies, and HRP-conjugated streptavidin. Chemiluminescent signals were recorded with a Chemidoc system. (**D**) To monitor virus-mediated cell-cell fusion, NiV-infected cells were fixed at 24 h p.i. and syncytia were visualized by crystal violet staining. Images of the whole well (left) and representative magnified images (right) are shown. (**E**) Total number of syncytia in a well. Error bars indicate SD. (**F**) Syncytium sizes were measured using ImageJ. Statistical differences were examined by unpaired *t*-test. Not significant (ns), *P* > 0.05. Asterisks indicate *P* values (*, *P* ≤ 0.05; **, *P* ≤ 0.01; ****, *P* ≤ 0.0001).

We found that Tg treatment significantly reduced viral titers in the supernatants of NiV infected cells (Fig. 1A). In cell lysates, NiV N mRNA levels measured by qPCR (Fig. 1B) and the amounts of viral proteins (Fig. 1C) were also massively reduced. This potent inhibitory effect also led to a decreased cell-cell fusion (Fig. 1D). While the total number of syncytia was not reduced (Fig. 1E), syncytium sizes were strongly decreased (Fig. 1F) indicating that Tg did not influence NiV entry but later replication steps such as RNA and/or protein synthesis. Taken together, these results indicate that sustained and irreversible ER stress and UPR activation as induced by Tg, had a pronounced antiviral effect.

### NiV glycoproteins induce the IRE1/XBP1 axis of the UPR

**Fig 2:**
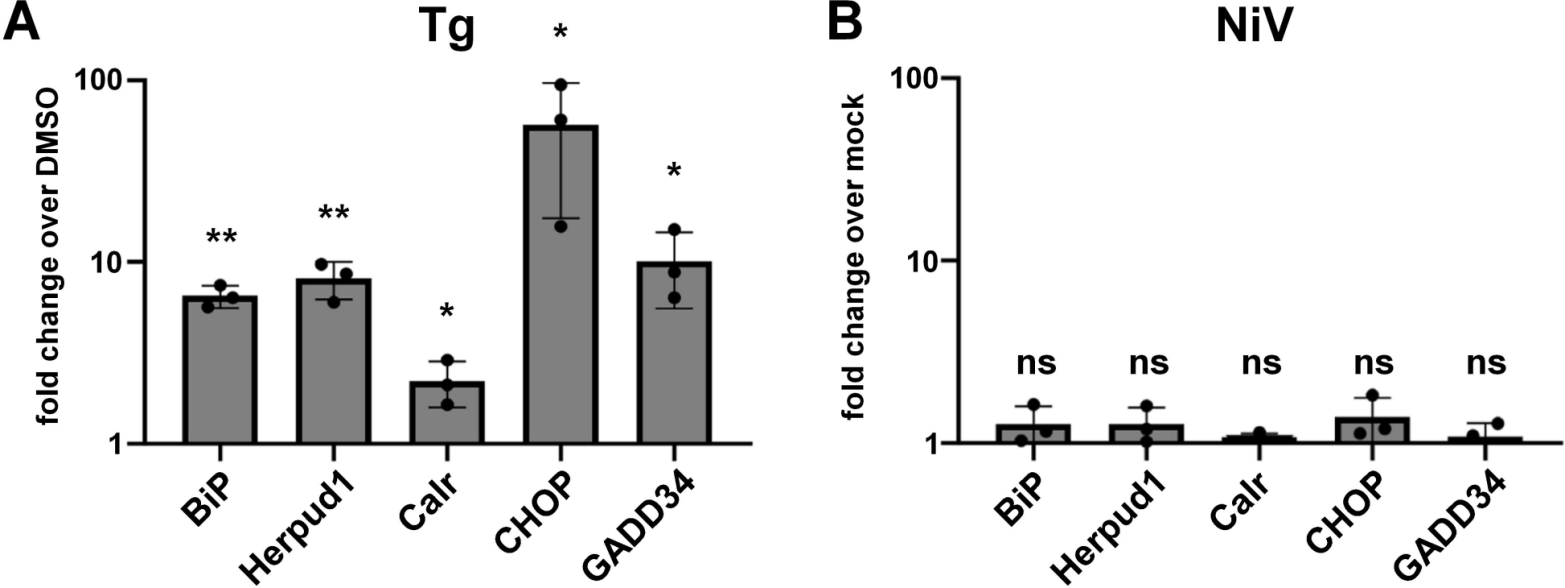
UPR target gene upregulation in Tg-treated and NiV-infected cells. (**A**) Vero76 cells were incubated without (DMSO control) or with 500 nM thapsigargin (Tg). After 16 h, total RNA was isolated from cell lysates and reverse transcribed using oligo(dT) primers. cDNA was then analyzed by quantitative real-time PCR using specific primers for the IRE1/XBP1 target genes BiP and Herpud1, the ATF6 target gene calreticulin (Calr) and the PERK pathway target genes CHOP and GADD34. Ct values were normalized to the internal control (RPS 18) and the fold change in mRNA levels relative to DMSO-treated cells was calculated (2^−ΔΔct^). (**B**) Cells were infected with NiV at a MOI of 0.1 for 18 h. Then, RNA was extracted and analyzed by quantitative RT-PCR as described above. The fold change in mRNA levels relative to mock-infected controls is shown (2^−ΔΔct^). Data are represented as mean ± SD. To determine the statistical significant differences from the baseline expression, one sample *t* test against 0 was performed using the ΔΔct values. Asterisks indicate *P* values (*, *P* ≤ 0.05; **, *P* ≤ 0.01). Not significant (ns), *P* > 0.05.

The finding that sustained ER stress inhibited NiV infection raised the question of how NiV ensures productive replication despite the massive synthesis of NiV glycoproteins in the ER, which must be assumed to at least activate the major IRE1/XBP1 branch of the UPR, as is the case for other viral glycoproteins (16, 25, 30). To address this question, we investigated the UPR induction in NiV-infected cells and in control cells treated with Tg by quantitating the upregulation of representative IRE1-, ATF6-, or PERK-dependent target genes (31). As expected, sustained and broad UPR induction by Tg led to a significant upregulation of all genes tested (Fig. 2A). In contrast to Tg, none of the UPR target genes were upregulated in NiV-infected cells at 18 h p.i. (Fig. 2B). As in Vero76 cells, Tg treatment also induced a pronounced UPR gene upregulation in human cerebral microvascular endothelial and lung epithelial cells (hCMEC/D3 and A549 cells), while these UPR target genes were not induced by NiV at 24 h p.i. (Fig. S1).

The lack of a detectable ER stress response in NiV-infected cells at a time point of infection when viral replication, protein synthesis, and cell-cell fusion have been ongoing for many hours raised the question of whether the synthesis of the NiV glycoproteins might not trigger an UPR response at all.

**Fig 3:**
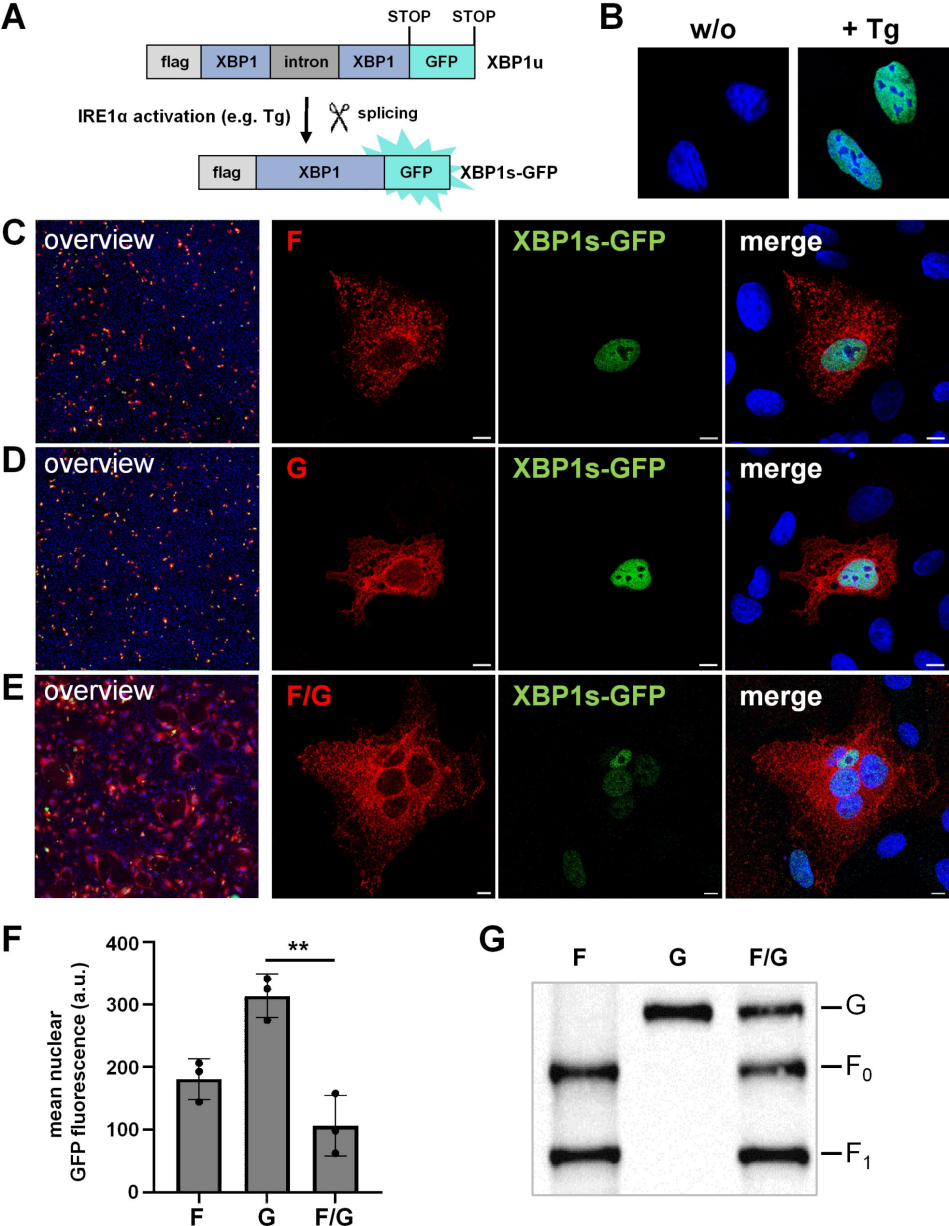
Induction of XBP1 splicing by the NiV glycoproteins. (**A**) Scheme of the pCAGGS-XBP1u-GFP reporter plasmid. UPR activation via IRE1α leads to XBP1u mRNA splicing and expression of the fusion protein XBP1s-GFP, which translocates to the nucleus. (**B**) Nuclear XBP1s-GFP expression in cells incubated without (w/o) or with 500 nM Tg (+Tg). (**C–E**) Vero76 cells were transfected with the XBP1u-GFP reporter plasmid and plasmids encoding HA-tagged NiV F (**C**), HA-tagged NiV G (**D**), or both F/G glycoproteins (**E**). At 20 h p.t., cells were fixed with PFA and permeabilized with methanol/acetone. Glycoproteins (red) were detected with an HA-specific antibody. XBP1s-GFP (green) was detected by autofluorescence. Nuclei were counterstained with DAPI (blue). Left panels show merged overview images acquired by widefield (non-confocal) fluorescence microscopy using a 20× objective. Right panels present confocal fluorescence images at higher magnification, illustrating NiV glycoprotein and XBP1s-GFP signals in representative F-, G-, or F/G-expressing cells. Scale bars: 10 µm. (**F**) To quantify XBP1 splicing, the mean nuclear XBP1s-GFP fluorescence in NiV glycoprotein-positive cells or syncytia was determined (a.u., arbitrary units) and is presented as mean ± SD of three independent experiments (>5,000 cells per replicate). Statistical analysis was conducted by a one-way ANOVA and Tukey’s post-hoc test (**, *P* ≤ 0.01). (**G**) To control NiV glycoprotein expression, lysates from cells expressing HA-tagged F and/or G proteins were subjected to SDS-PAGE and western blot analysis using HA-specific antibodies. A representative blot of three individual experiments is shown (*n* = 3).

To address this question, we investigated whether the NiV glycoproteins, like many other viral glycoproteins (16, 24, 25, 32), activate the most conserved and major axis of the UPR, the IRE1/XBP1 pathway. To determine whether one or both NiV glycoproteins can induce expression of the transcription factor XBP1s, the key factor in alleviating ER stress (31, 33, 34), we used an XBP1u-GFP reporter plasmid (25). Transcription of the reporter plasmid produces unspliced XBP1u mRNA, which in the absence of UPR activation, leads to the expression of a non-fluorescent XBP1u reporter protein. Upon ER stress and IRE1 activation, the XBP1u mRNA is spliced. The resulting frameshift leads to the generation of a spliced XBP1s mRNA encoding a fluorescent XBP1s-GFP fusion protein, which, after translation, is translocated to the nucleus (Fig. 3A). This allows to detect UPR activation and XBP1 splicing by monitoring nuclear XBP1s-GFP signal, as shown in Tg-treated cells expressing the XBP1u-GFP reporter plasmid (Fig. 3B). To monitor the activation of the IRE1/XBP1 pathway by the two NiV glycoproteins, cells transfected with the XBP1u-GFP reporter plasmid and HA-tagged NiV F or G protein were fixed at 20 h p.t. and immunostained with HA-specific antibodies. Like Tg, the expression of either NiV F or NiV G induced IRE1 activation and XBP1 splicing, as shown by nuclear XBP1s-GFP expression in NiV glycoprotein positive cells (Fig. 3C and D). Surprisingly, upon coexpression of NiV F and G (F/G), which resulted in cell-cell fusion and syncytium formation, nuclear XBP1s-GFP expression was less pronounced (Fig. 3E). Quantification of the XBP1 splicing activity (mean nuclear XBP1s-GFP fluorescence) in cells expressing NiV F or G or both glycoproteins (Fig. 3F) clearly confirmed the more limited UPR activation/XBP1 splicing upon NiV glycoprotein coexpression and cell-cell fusion. Since the glycoprotein content did not differ in F- or G- and F/G-coexpressing cell cultures (Fig. 3G), the limited UPR activation in cells expressing both NiV glycoproteins could not be explained by reduced viral protein expression. This, together with the finding that nuclear XBP1s-GFP expression was even more reduced in larger syncytia at later time points (Fig. S2), suggested that F/G-mediated cell-cell fusion may play a role in regulating the UPR.

### Cell-cell fusion limits NiV glycoprotein-induced XBP1 splicing

**Fig 4:**
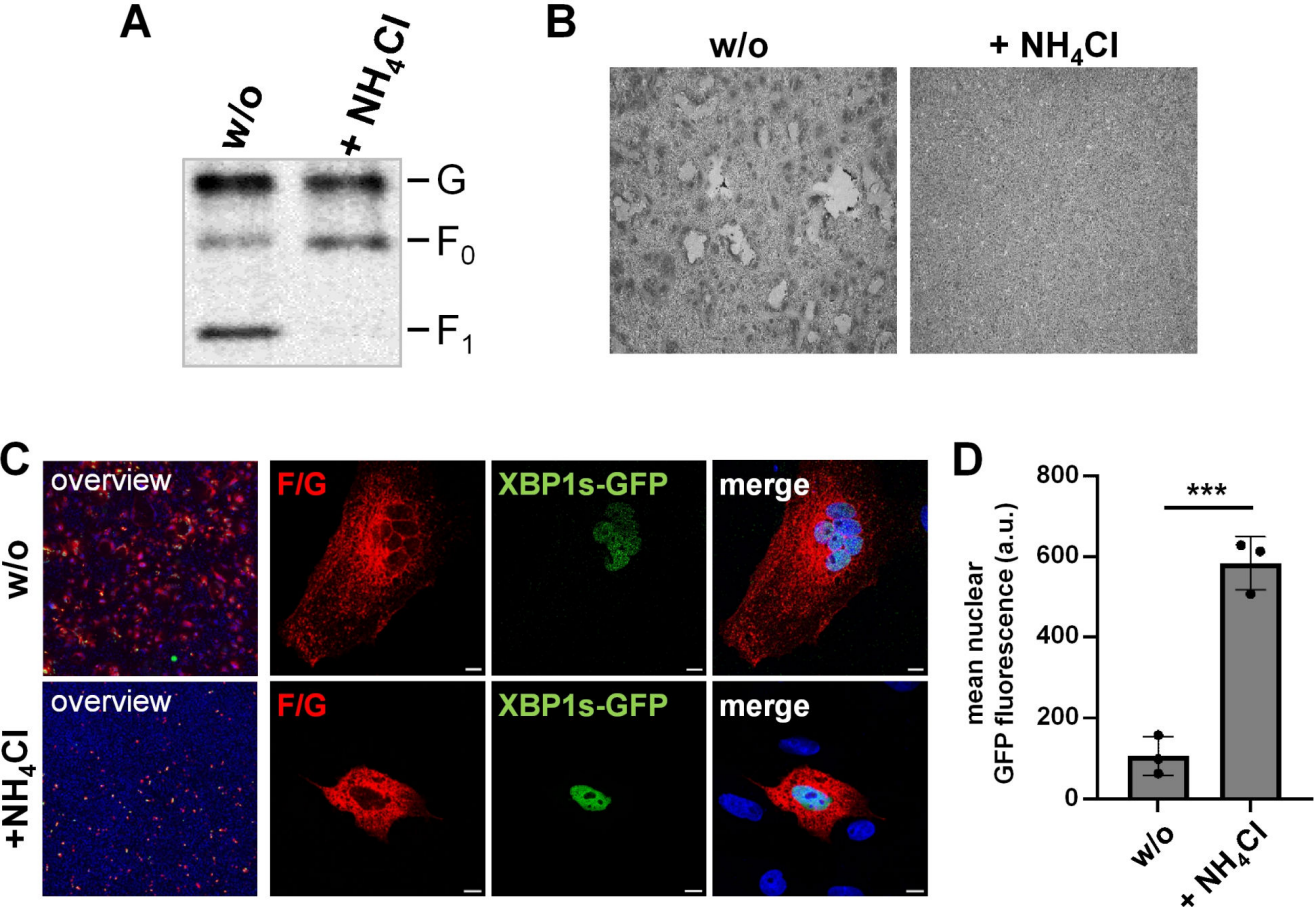
XBP1 splicing is limited in the presence of syncytium formation. (**A**) Vero76 cells were transfected with plasmids encoding HA-tagged F and G. 4 h p.t. 20 mM NH_4_Cl was added to the respective samples for 16 h. Cells were lysed and subjected to western blot analysis. NiV G and F were detected with an HA-specific antibody. One representative blot of three individual experiments (*n* = 3) is shown. (**B**) Cell monolayers similarly treated to (**A**) were fixed and stained with Giemsa staining solution. (**C**) Vero76 cells were transfected with plasmids encoding the XBP1u-GFP reporter, HA-tagged NiV F and G proteins and were treated without (w/o) or with 20 mM NH_4_Cl at 4 h p.t.. 20 h p.t. cells were fixed for immunofluorescence. Glycoproteins (red) were visualized with an HA-specific antibody. XBP1s-GFP (green) was detected by autofluorescence. Nuclei were counterstained with DAPI (blue). As described in the legend to Fig. 3, widefield (non-confocal) overview images and confocal images of representative F/G-expressing cells are shown. The images of untreated F/G-expressing cells (w/o) in this figure and in Fig. 3E were obtained from the same experimental sample. Scale bars: 10 µm. (**D**) Quantification of XBP1 splicing was performed as described in the legend to Fig. 3. Data are represented as mean nuclear XBP1s-GFP fluorescence ± SD, and statistical analysis was conducted using an unpaired *t*-test. ***, *P* ≤ 0.001.

To evaluate the idea that cell-cell fusion is responsible for the limited UPR activation in cells coexpressing NiV F and G, we inhibited syncytium formation using ammonium chloride (NH_4_Cl). This lysosomotropic weak base raises the pH in acidic vesicles and blocks NiV cell-cell fusion by preventing pH-dependent F_0_ cleavage into fusion active F_1/2_ by cathepsin L/B in endosomes (12, 13, 35). To control the functional effect of the NH_4_Cl treatment, Vero76 cells expressing HA-tagged NiV F and G were cultivated in the absence and presence of 20 mM NH_4_Cl. After 20 h, F cleavage and syncytium formation were analyzed by western blot analysis or Giemsa staining, respectively. As expected, treatment with NH_4_Cl did not affect NiV G or F expression but inhibited F_0_ cleavage, as indicated by the lack of the F_1_ cleavage product (Fig. 4A, + NH_4_Cl). In line with the lack of fusion-active F proteins, syncytium formation was not observed in NH_4_Cl treated cells (Fig. 4B). To analyze the effect of fusion inhibition on NiV F/G induced XBP1 splicing, Vero76 cells were transfected with the XBP1u-GFP reporter plasmid and plasmids encoding HA-tagged NiV F and G. At 4 h p.t., the cells were treated with 20 mM NH_4_Cl and the expression of nuclear XBP1s-GFP was analyzed as an indicator for UPR activation. As observed before, untreated cells showed syncytium formation with limited nuclear GFP signals (Fig. 4C, w/o). In contrast, if cell-cell fusion was blocked, nuclear XBP1s-GFP expression was consistently detected in NiV F/G expressing cells (Fig. 4C, + NH_4_Cl). This was also the case in F/G-expressing A549 cells (Fig. S3), or when syncytium formation was blocked by neutralizing antibodies instead of adding NH_4_Cl (Fig. S4A). Quantification of the mean nuclear XBP1s-GFP expression in the NiV glycoprotein expressing cell populations (Fig. 4D, Fig. S4B) clearly supported the conclusion that UPR activation by the NiV glycoproteins is limited if syncytium formation can occur, whereas blocking cell-cell fusion leads to increased ER stress and XBP1u splicing.

### Cell-cell fusion and UPR activation negatively correlate

**Fig 5:**
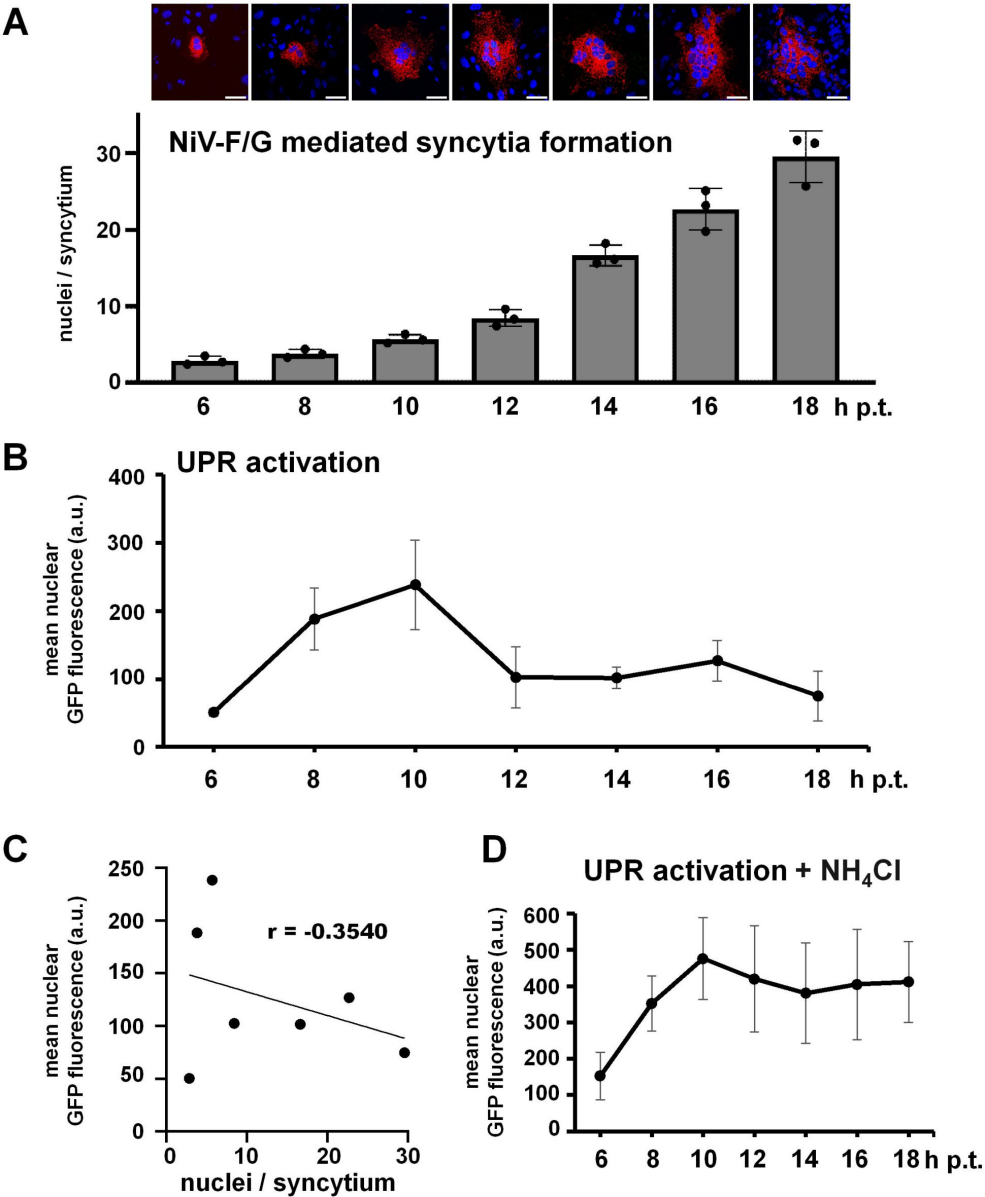
Syncytium formation limits XBP1 splicing. (**A**) Vero76 cells seeded on coverslips were transfected with plasmids encoding the XBP1u-GFP reporter and HA-tagged NiV F and G glycoproteins. At the indicated time points, cells were fixed and NiV glycoprotein positive syncytia (red) were detected by immunostaining as described in the legend to Fig. 3. Immunofluorescence images show representative syncytia at each time point. Scale bars: 50 µm. Syncytium sizes (nuclei/syncytium) of 30 randomly chosen syncytia of three independent experiments were determined for each time point and are shown as mean ± SD. (**B**) Mean XBP1s-GFP fluorescence in the nuclear region of NiV glycoprotein positive syncytia was assessed at indicated time points as described in the legend to Fig. 3 and is shown as mean ± SD. (**C**) Correlation of mean XBP1 splicing (*y*-axis) and mean syncytium size (*x*-axis). The trend line for the linear regression and the calculated Pearson correlation coefficient *r* are shown in the scatter plot. (**D**) At 6 h p.t., Vero76 cells expressing the XBP1-GFP reporter and HA-tagged NiV F and G glycoproteins were treated with 20 mM NH_4_Cl to block fusion. XBP1 splicing in the absence of syncytium formation was quantified as described before.

Since syncytium formation and UPR activation are dynamic processes (15, 36), we aimed to monitor the correlation of XBP1u splicing and syncytium formation over time. For this, Vero76 cells expressing the XBP1u-GFP reporter and HA-tagged F and G were fixed at different time points (6–18 h p.t.) and monitored for syncytium formation and nuclear XBP1s-GFP expression. Quantification of cell-cell fusion (nuclei per syncytium) showed that syncytium sizes continuously increased over time (Fig. 5A). In contrast, UPR activation (nuclear XBP1s-GFP in NiV F/G positive cells) only increased until 10 h p.t., i.e., at time points when syncytium sizes were still very small (<5 nuclei/syncytium). After having reached a maximal fluorescent intensity of about 250 arbitrary units (a.u.), the mean nuclear XBP1s-GFP expression in NiV glycoprotein positive syncytia decreased again and stayed at a level of about 100 a.u. (Fig. 5B). The graph in Fig. 5C shows a negative correlation of syncytium sizes and UPR induction (XBP1u splicing). Supporting this correlation, no substantial decrease in nuclear XBP1s-GFP expression was observed when syncytium formation was inhibited by adding NH_4_Cl. Here, after reaching a maximum, the mean nuclear GFP signal remains on a level at 400–500 a.u. (Fig. 5D).

## DISCUSSION

Viruses hijacking the ER-associated protein synthesis machinery often induce adaptive host cell stress responses such as the IRE1/XBP1 pathway of the UPR to restore ER homeostasis. This initially allows the cell to survive the inordinate stress of *de novo* synthesis of high amounts of viral proteins by significantly increasing the ER folding capacity. However, if ER stress is not resolved by this cytoprotective UPR response, apoptosis can be initiated, which counteracts viral replication and spread. Thus, UPR activation represents a time-dependent pro- or antiviral factor that can influence the pathogenesis and virulence of a viral infection (16, 37). In this study, we showed that sustained induction of all UPR branches by thapsigargin drastically reduced viral RNA and protein synthesis, NiV titers, and syncytium formation, demonstrating that extensive ER stress and irreversible activation of the UPR had clear antiviral effects. Like many other viral glycoproteins, the NiV glycoproteins activate the IRE1/XBP1 branch of the UPR although the induction appears to be temporally controlled. Our data suggest that the UPR activation and XBP1s expression are initially induced, likely to increase the ER folding capacity and support the production of correctly folded and processed F/G glycoproteins. These are subsequently expressed on the cell surface and limit further UPR activation by cell-cell fusion leading to glycoprotein and ER stress dilution. If syncytium formation is blocked, UPR activation in NiV glycoprotein-expressing cells continues to increase. The finding that UPR activation negatively correlates with syncytium size indicates that NiV utilizes cell-cell fusion as a way to limit the potential antiviral effects of excessive and sustained glycoprotein-induced UPR activation.

Given the critical role of the UPR in viral replication, recent studies have indicated that the ER stressor Tg exerts a potent antiviral effect against RNA viruses, including influenza and coronaviruses. Inhibition by Tg has also been reported for several viruses of the order *Mononegavirales*, such as Marburg virus, respiratory syncytial virus, or Newcastle disease virus (29, 38–40). The present study, which shows that the replication of NiV was also potently inhibited by this chemical ER stress inducer, expands the group of Tg-sensitive viruses and underlines the potential of Tg and its derivatives as broad-spectrum antivirals (41).

**Fig 6:**
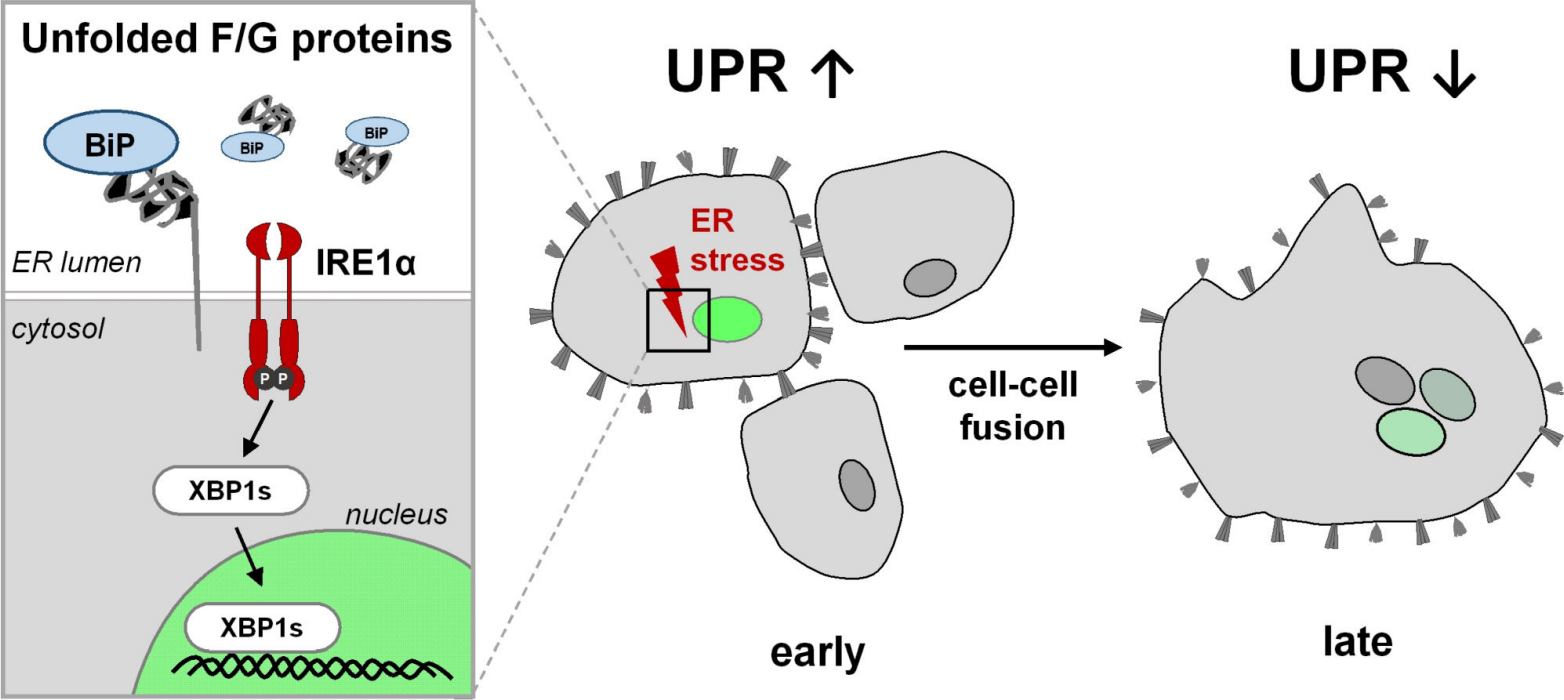
Model for UPR activation and regulation by NiV-mediated cell-cell fusion. Expression of NiV F and NiV G glycoproteins initially induces ER stress due to the accumulation of unfolded proteins and the recruitment of ER chaperones (e.g., BiP). This activates the IRE1α/XBP1 axis of the UPR to increase the ER folding capacity in early infection phases. If sufficient correctly folded and fusion-active glycoproteins have been produced and transported to the plasma membrane, cell-cell fusion with neighboring cells is initiated. Because of the syncytium formation, viral glycoprotein synthesis is no longer confined to the initially infected cell, thereby reducing ER stress per cell and limiting UPR activation. This allows the production of large quantities of viral glycoproteins while preventing detrimental ER stress potentially leading to UPR-related apoptosis that could limit viral replication and spread.

In contrast to Tg, which globally activates the UPR and effectively induced CHOP and GADD34 expression, key components of the PERK branch of the UPR responsible for translational shutdown and apoptosis (16, 42), these UPR target genes were not upregulated in NiV-infected cells at 18 h p.i.. This suggests that NiV has evolved a way to avoid triggering potentially maladaptive UPR outcomes although the synthesis of its viral glycoproteins caused ER stress and induction of the IRE1/XBP1 pathway. This selective activation of the UPR likely supports proper folding of the viral glycoproteins, a strategy that has also been proposed for influenza A virus and tick-borne encephalitis virus glycoproteins (24, 43, 44). The initial NiV F/G-mediated ER stress leading to XBP1 mRNA splicing likely supports the restoration of the ER homeostasis allowing a timely expression of functional NiV glycoproteins on the cell surface, a prerequisite for infectious virus particle assembly and cell-cell fusion. The latter not only functions as a way to spread NiV infection within cell layers or tissues (15) but also serves as a regulatory mechanism to limit UPR activation by preventing sustained and unresolved ER stress despite ongoing viral glycoprotein synthesis. Figure 6 depicts our model suggesting that UPR activation in early infection phases is required to increase the ER folding capacity to support the expression of functional NiV glycoproteins, which then prevent further increasing UPR activation with potential antiviral effects by inducing cell-cell fusion. The finding that UPR can be induced by Tg when syncytia have already formed (Fig. S5) strengthens the idea that cell-cell fusion limits or dilutes UPR rather than preventing its onset.

By regulating the UPR through syncytium formation, NiV has evolved a strategy to limit UPR activation. Though it remains to be determined how fusion exactly mitigates ER stress and if there are additional compensatory or regulating effects of the ATF6 and PERK branches of the UPR, the finding that XBP1 splicing was also reduced in syncytia formed by the measles virus glycoproteins (Fig. S6) provides initial evidence that NiV might not be the only virus that limits UPR through cell-cell fusion. This as yet undescribed strategy complements the mechanisms of UPR modulation developed by viruses such as cytomegalovirus, coronaviruses or Marburg virus to maintain beneficial aspects of the UPR and suppress deleterious ones (22, 25, 45).

The ER is a highly dynamic organelle, continuously undergoing rearrangements that include tubule branching and ER partitioning. These dynamic changes in ER morphology allow flexible physical adaptation to stress. The ER also has functional connections to other cellular organelles, with the plasma membrane associated ER sites supposed to be involved in phosphatidylinositol metabolism, non-vesicular transfer of sterols, and Ca^2+^-level regulation (46). These functionally important ER-plasma membrane contact sites, which are stabilized by proteins tethering the two membranes, are highly dynamic and constantly remodeled, for example during cell division (47). Considering that the formation of syncytia, like mitotic processes, leads to massive changes in cell shape and causes rearrangements in the plasma membrane, NiV-mediated cell-cell fusion probably also triggers dynamic ER remodeling to meet the altered needs of the cell. These adaptations may allow the redistribution of ER stress-inducing proteins (NiV glycoproteins) across the expanded ER volume of fused cells. Continued cell-cell fusion and the resulting increase in ER capacity could subsequently help to mitigate further ER stress and limit the UPR. This idea is supported by our finding that no reduction in UPR activation was observed if syncytium formation was blocked and NiV glycoprotein expression, and thus ER stress, was confined to a single cell.

NiV has a broad cell tropism *in vitro* and *in vivo*. Epithelial and microvascular endothelial cells in lung, spleen, and kidney are the major cell types infected by NiV *in vivo*, with infection of microvascular endothelial cells in the CNS being regarded as the major basis for the development of NiV encephalitis (48, 49). Syncytium formation is not only observed in NiV-infected endothelial cells cultures (Fig. S7) but also found in histopathological specimens from natural or experimental infections (48, 50). The fact that syncytium formation is frequently observed in NiV infections both *in vitro* and *in vivo* (15) underlines the potential importance of cell-cell fusion-mediated UPR regulation for productive NiV infection. However, quantitative differences across various cell types and tissues have been noted, likely due to cell-specific variations in NiV receptor expression or other host factors such as membrane cholesterol (51). These variations in syncytium formation would be expected to influence fusion-dependent UPR regulation, which, in addition to affecting the direct spread of NiV between cells in the absence of particle release, may play a crucial role in determining the efficiency of NiV replication in different cell types.

In summary, this study provides first evidence that syncytium formation reduces or dilutes the NiV glycoprotein-induced ER stress to prevent sustained and potentially antiviral UPR activation. This suggests that NiV-induced syncytium formation is not only an important way to mediate viral spread from cell to cell but also limits activation of cellular stress responses. Since syncytium formation is also induced by other viruses (15, 52, 53), studies on the influence of syncytium formation on ER stress upon infection with fusogenic viruses could provide valuable insight regarding the dynamic interplay of viral and host cell factors, and the extent cell-cell fusion contributes to the complex modulation of cellular stress responses.

## MATERIALS AND METHODS

### Virus infection

Infection experiments with NiV were conducted under biosafety level 4 (BSL-4) conditions at the Institute of Virology, University Marburg. The NiV_Malaysia_ (NiV) isolate that was used for this study has been described previously (54). Vero76 cells (CRL-1587, ATCC) were cultivated in Dulbecco’s modified Eagle’s medium (DMEM, Gibco) with 10% fetal calf serum (FCS, Gibco), 100 U penicillin mL^−1^ (Gibco), 0.1 mg streptomycin mL^1^ (Gibco), and 4 mM L-glutamine (Gibco). Confluent Vero76 cells grown in 12-wells (5 × 10^5^ cells) or on cover glasses in 24-wells (2.5 × 10^5^ cells) were infected with NiV at an MOI of 0.1 for 1 h at 37 °C. After virus adsorption, cells were washed five times with DMEM 2% FCS and incubated in DMEM 2% FCS containing 500 nM thapsigargin (Tg; Sigma), 20 mM NH_4_Cl (Merck), or DMSO (Wak-Chemie) for 18–24 h. To determine infectious viral titers, the cell-free supernatants of infected cells were collected and quantified by serial dilution on Vero76 cells. 50% tissue culture infectious dose (TCID_50_/mL) was calculated using the Reed-Muench method.

### Plasmids and transfection

pCG and pCAGGS-vector based expression plasmids encoding NiV-M, HA-tagged NiV-F, and HA-tagged NiV-G have been described previously (13, 55). The reporter plasmid pCAGGS-XBP1-GFP was provided by C. Rohde (25). Cells at 80% confluency were transfected using Lipofectamine 2000 (Invitrogen) or TransIT (Mirus Bio) according to the manufacturer’s protocol. At 4–6 h post transfection (p.t.), the medium was exchanged with DMEM 10% FCS. If applicable, media contained 500 nM of Tg, DMSO, or 20 mM NH_4_Cl.

### Antibodies and reagents

The following antibodies were obtained commercially: NiV N (Alpha Diagnostic, NiV21-A), NiV F (AntibodySystem, PVV08101), NiV G (AntibodySystem, PVV07901), HA-tag (Sigma, H6908), biotinylated anti-rabbit IgG (Cytiva, RPN1004V), biotinylated anti-mouse IgG (Cytiva, RPN1001V), peroxidase-conjugated streptavidin (Cytiva, RPN1051V), Alexa Fluor 568-conjugated anti-rabbit IgG (Invitrogen). Polyclonal anti-NiV guinea pig serum (gp3) was kindly provided by H. Feldmann. Polyclonal anti-NiV M rabbit serum (IG1321) was produced by ImmunoGlobe. Nuclei were stained with 4,6-diamidino-2-phenylindole (DAPI, Invitrogen).

### Immunofluorescence microscopy

Immunofluorescence staining was performed as described previously (56). Vero76 cells grown on glass coverslips were fixed at indicated time points with 4% paraformaldehyde (PFA, Merck). PFA was quenched with 0.1 M glycine (Roth); cells were permeabilized with ice-cold 1:1 methanol (Sigma)/acetone (Roth) and incubated for 1 h in blocking buffer (2% BSA (Serva), 5% glycerol (Roth), 0.2% Tween20 (Sigma), and 0.05% NaN3 (Merck). After staining with primary antibodies and incubation with Alexa Fluor-conjugated secondary antibodies for 1 h, coverslips were embedded in mowiol (Calbiochem). Confocal images were acquired on a Leica TCS SP5 II or Stellaris 8 confocal laser scanning microscope with a 63×/1.4 NA oil objective. For quantification, widefield fluorescence images were captured on a Leica Thunder Cell Imager using a 20×/0.40 NA objective to generate large tile scans containing more than 5,000 cells per sample. ImageJ (https://imagej.net/ij) was used to define ROIs containing the glycoprotein-expressing cells (Alexa Fluor 568 channel). Nuclei in these ROIs were identified using the DAPI channel and average GFP intensity within the nuclear area was measured after background-subtraction (GFP channel). The mean nuclear GFP fluorescence intensity is given in arbitrary units (a.u.) and served as a quantitative measure of UPR activation.

### Crystal violet and Giemsa staining

NiV-infected cells were fixed with 4% PFA for 48 h and stained with 0.1% crystal violet (Sigma). The total number of syncytia was counted manually using a light microscope (AMG AMEX 1100 Cell Imager). For quantification of syncytium sizes, complete wells (*n*=3) were imaged using the Bio-Rad ChemiDoc Touch Imaging System and the area covered by syncytia was analyzed by ImageJ.

To visualize syncytium formation in transfected cells, Vero 76 cells coexpressing the NiV glycoproteins were fixed with ethanol at 20 h post transfection and stained with 1:10 diluted Giemsa staining solution (Sigma) for 30 min.

### SDS-PAGE and western blot

Cells were lysed with RIPA buffer (1% Triton (Roth), 1% deoxycholic acid (Sigma), 0.1% sodium dodecyl sulfate (SDS, Roth), 0.15 M NaCl (Roth), 20 mM Tris/HCl pH 7.5 (Roth, Merck), 10 mM EDTA (Sigma) containing 1:25 cOmplete protease inhibitor (Merck). NiV-infected cells (samples from the BSL-4 facility) were harvested and inactivated in phosphate-buffered saline (PBS) containing 1% SDS and heated two times for 10 min at 100°C. Lysates were then mixed with 2× sample buffer (20% glycerol (Roth), 100 mM Tris pH 6.8 (Roth), 0.04% bromophenol blue (Merck), 3-4% SDS (Roth), 4% β-mercaptoethanol (Sigma), heated for 10 min at 100°C, and subjected to SDS-PAGE under reducing conditions. Proteins were transferred to nitrocellulose membranes. After blocking with 5% skim milk, membranes were incubated with primary antibodies followed by staining with biotinylated secondary antibodies and horseradish peroxidase (HRP)-conjugated streptavidin. Chemiluminescence was detected with SuperSignal West Dura substrate (ThermoFisher) using the BioRad ChemiDoc Touch Imaging System. Quantification was performed using the ImageLab software (BioRad).

### Quantitative real-time polymerase chain reaction

Total RNA was isolated using the RNeasy Kit (Qiagen) and reverse-transcribed with oligo(dT)_18_ primers using the RevertAid First Strand cDNA Synthesis Kit (ThermoFisher) following the manufacturer’s instructions. PCRs were performed using 50 ng of cDNA together with 2× Maxima SYBR Green qPCR Master Mix (ThermoFisher) and 60 pmol of the respective forward and reverse primers in a total volume of 25 µL. Amplification was carried out using a StepOne Real-Time PCR System (Applied Biosystems) or a qTOWER³ (Analytik Jena) as follows: 10 min at 95°C, followed by 40 cycles of 15 s at 95°C, 15 s at 53°C, and 30 s at 72°C. Specificity of the amplification was confirmed using a melting curve analysis. To determine mRNA expression levels, ct values were normalized to the internal housekeeping gene by subtraction (Δct) and represented as 2^−Δct^ or additionally normalized to the untreated/uninfected control (ΔΔct) and represented as 2^−ΔΔct^ (fold change over untreated/mock). Gene-specific primers were custom-synthesized and purchased from Eurofins Genomics. The following primers were used in this study: tubulin for GGCCGTGTTTGTAGACTTGG, tubulin rev CTTCCTTGCCTGTGATGAGC, NiV N for ATCAATCGTGGTTATCTTGA, NiV N rev CAGCCAGTTCTGCAACTTGATC, RPS18 for GCGGCGGAAAATAGCCTTTG, RPS18 rev GATCACACGTTCCACCTCATC, GADD34 for AAACACTGGGCCTGAAAACCA, GADD34 rev GCTGGTTGCTTCTTGCTCACT, Calreticulin for GAGCAGAACATCGACTGTGGG, Calreticulin rev GGCCACAGATATCAGGACCAAA, CHOP for CTCCTGGAAATGAAGAGGAAGAATC, CHOP rev GCTTGTGACCTCTGCTCGTT, Herpud1 for AACGGCATGTGTTGCATCTGGT, Herpud1 rev CTGTGGATTCAGCCACCTTGG, BiP for AGGCTTATTTGGGAAAGAAGGTTAC, BiP rev GATCCTCATAACATTTAGGCCAGC.

### Quantification and statistical analysis

GraphPad Prism Software was used for statistical analysis and figure generation. Statistical tests used are indicated in the respective figure legends. Asterisks indicate statistical significance ∗ *P* ≤ 0.05; ∗∗ *P* ≤ 0.01; ∗∗∗ *P* ≤ 0.001; ∗∗∗∗ *P* < 0.0001.

## Data Availability

All data supporting the findings of this study are available within the article. Further details can be obtained from the corresponding author.

## SUPPLEMENTAL MATERIAL

Supplemental figures jvi.01046-25-s0001.pdf Figures S1 to S7.

ASM does not own the copyrights to Supplemental Material that may be linked to, or accessed through, an article. The authors have granted ASM a non-exclusive, world-wide license to publish the Supplemental Material files. Please contact the corresponding author directly for reuse.
