# Supplementary material for: Cell-cell fusion limits activation of the unfolded protein response induced by the Nipah virus glycoproteins
Source: J Virol. 2025 Dec 11;100(1):e01046-25. doi: 10.1128/jvi.01046-25 (PMC12817901; doi:10.1128/jvi.01046-25)
Supplement: Supplemental figures — Figures S1 to S7. [file jvi.01046-25-s0001.pdf]

Supplemental material for:

Cell-cell fusion limits activation of the unfolded protein response induced by the Nipah virus glycoproteins

by

Paula Jordan, Sören Heyer,
Julian Hüther, Ilka Fischer, Nico Becker, Andrea Maisner

References cited in Fig. S1-S7

1. Weksler B, Romero IA, Couraud PO. 2013. The hCMEC/D3 cell line as a model of the human blood brain barrier. Fluids Barriers CNS 10:16.
2. Schmidt R, Beltzig LC, Sawatsky B, Dolnik O, Dietzel E, Krähling V, Volz A, Sutter G, Becker S, von Messling V. 2018. Generation of therapeutic antisera for emerging viral infections. NPJ Vaccines 3:42
3. Moll M, Klenk HD, Maisner A. 2002. Importance of the Cytoplasmic Tails of the Measles Virus Glycoproteins for Fusogenic Activity and the Generation of Recombinant Measles Viruses. J Virol. 76:7174–7186.
4. Erbar S, Maisner A. 2010. Nipah virus infection and glycoprotein targeting in endothelial cells. Virol J. 7:305.
5. Gamble A, Yeo YY, Butler AA, Tang H, Snedden CE, Mason CT, Buchholz DW, Bingham J, Aguilar HC, Lloyd-Smith JO. 2021. Drivers and Distribution of Henipavirus-Induced Syncytia: What Do We Know? Viruses 13:1755.
6. Erbar S, Diederich S, Maisner A. 2008. Selective receptor expression restricts Nipah virus infection of endothelial cells. Virol J. 5:142.

Fig. S1

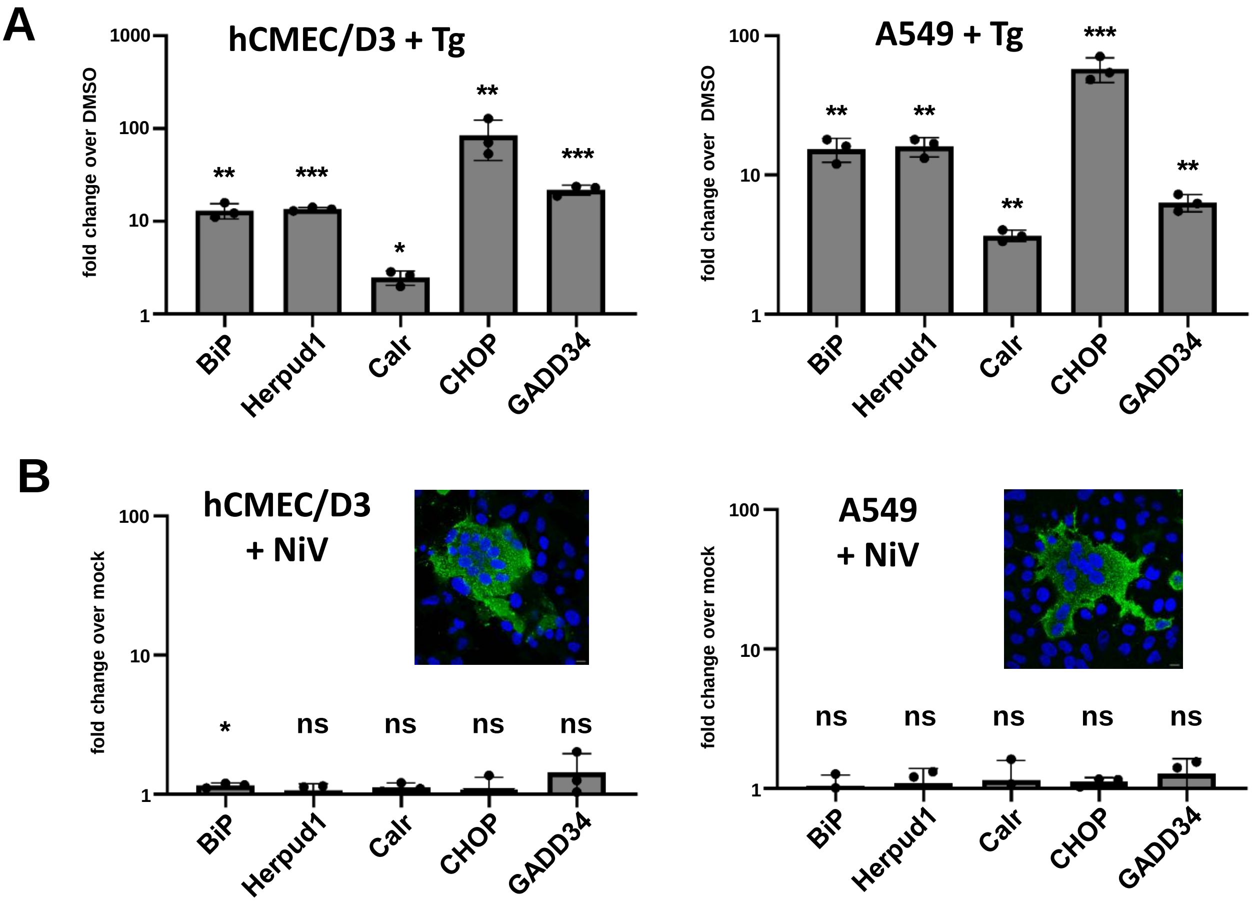

Figure S1. UPR target gene upregulation in Tg-treated and NiV-infected hCMEC/D3 and A549 cells. To validate the lack of UPR target gene induction in NiV-infected Vero76 cells shown in Fig. 2, two other cell types were analyzed. **(A)** Human cerebral microvascular endothelial cells (hCMEC/D3) [1] and lung epithelial cells (A549; ATCC CCL-185) were incubated without (DMSO control) or with 500 nM thapsigargin (Tg). After 16 h, total RNA was isolated from cell lysates and reverse transcribed using oligo(dT) primers. cDNA was then analyzed by qPCR using specific primers for the IRE1/XBP1 target genes BiP and Herpud1, the ATF6 target gene calreticulin (Calr) and the PERK pathway target genes CHOP and GADD34. Ct values were normalized to the internal control (RPS 18) and the fold change in mRNA levels relative to DMSO-treated cells was calculated ($2^{-\Delta\Delta ct}$). **(B)** Both cell lines were infected with NiV at a MOI of 10. At 24 h p.i., RNA was extracted and analyzed by quantitative RT-PCR as described above. The fold change in mRNA levels relative to mock-infected controls is shown ($2^{-\Delta\Delta ct}$). Data are represented as mean ± SD. To determine the statistical significant differences from the baseline expression, one sample t test against 0 was performed using the ΔΔct values. *, $p \leq 0.05$; **, $p \leq 0.01$; ***, $p \leq 0.001$. Not significant (ns). Inserts illustrate successful infection and syncytia formation in NiV-infected hCMEC/D3 and A459 cells detected by immunostaining at 24 h p.i..

As in Vero76 cells (see Fig. 2), Tg treatment induced a pronounced UPR upregulation in hCMEC/D3 and A549 cells, while UPR target genes were not induced by NiV at 24 h p.i..

Fig. S2

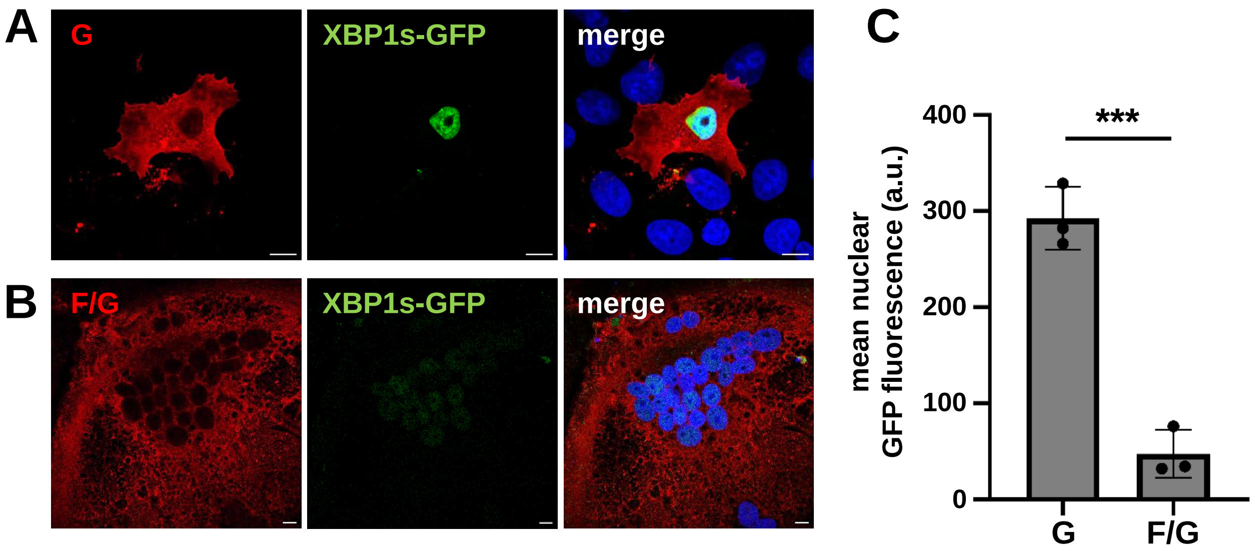

Figure S2. Induction of XBP1 splicing by the NiV glycoproteins at 44 h p.t..
(A, B) Vero76 cells expressing the XBP1u-GFP reporter plasmid and NiV G alone (A) or both NiV glycoproteins F and G (B) for 44 h. HA-tagged NiV glycoproteins (red) were stained with an HA-specific antibody and XBP1s-GFP (green) was detected by autofluorescence. Nuclei were counterstained with DAPI (blue). Scale bars: 10 µm. **(C)** Mean nuclear GFP fluorescence (XBP1 splicing) in the NiV glycoprotein expressing cell population was determined (arbitrary units, a.u.) and is shown as mean ± SD of three independent experiments (>5000 cells per replicate). Statistical analysis was conducted using an unpaired t-test. ***, p≤0.001.

XBP1 induction in very large syncytia at 44 h p.t. is even more reduced than in smaller syncytia formed at 20 h p.t. (see Fig. 3), which supports the idea that cell-cell fusion limits UPR activation.

Fig. S3

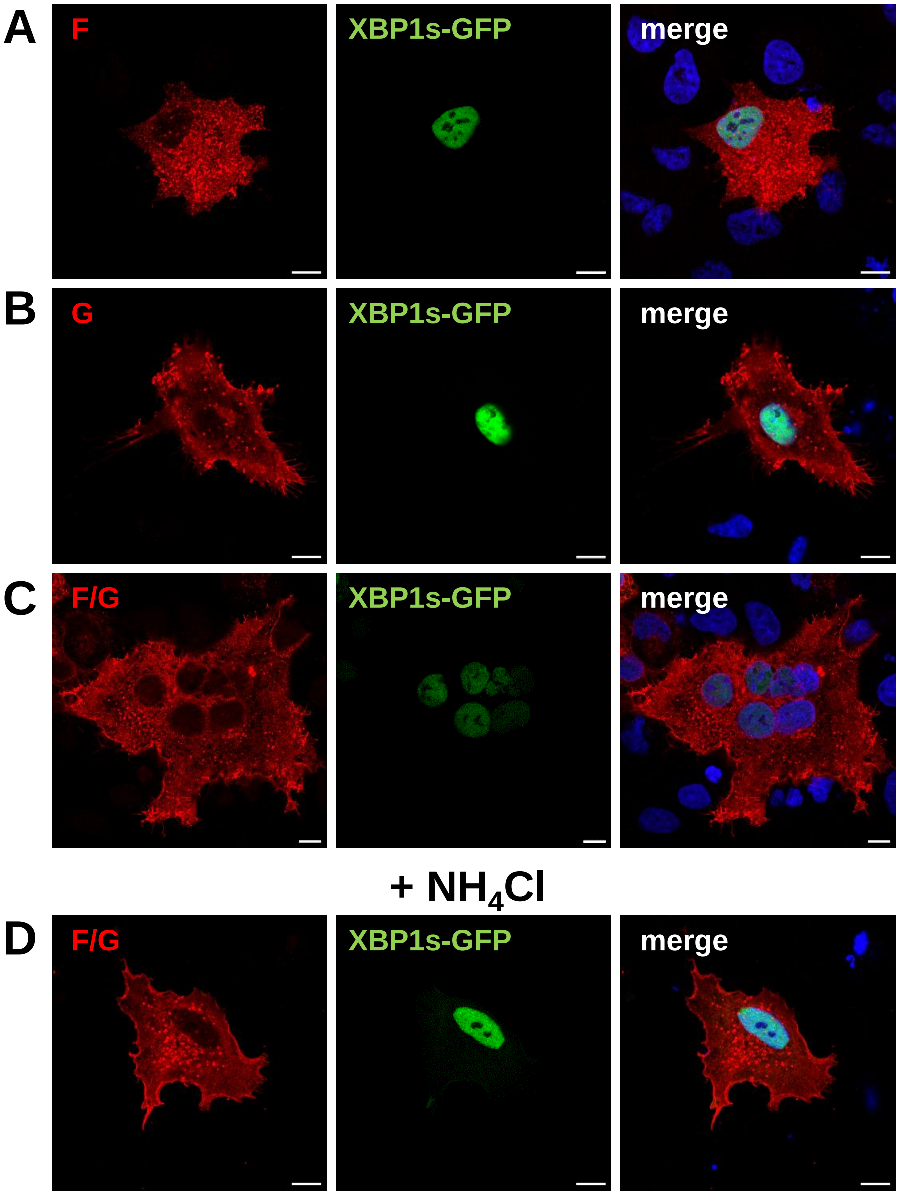

Figure S3. XBP1 splicing in A549 cells.
To analyze UPR activation by the NiV glycoproteins in another cell type, A549 cells were transfected with the XBP1u-GFP reporter plasmid and plasmids encoding HA-tagged NiV F **(A)**, HA-tagged NiV G **(B)** or both F/G glycoproteins **(C, D)**. To block syncytia formation in F/G-expressing cells, 20 mM NH_4Cl was added 4 h after transfection **(D)**. At 20 h p.t., cells were fixed with PFA and permeabilized with methanol/acetone. Glycoproteins (red) were detected with an HA-specific antibody. XBP1s-GFP (green) was detected by autofluorescence. Nuclei were counterstained with DAPI (blue). Scale bars: 10 µm.

Similar to what was found in Vero76 cells (see Fig. 3C-E), NiV F and G induced XBP1 splicing in A549 cells (A, B), while nuclear XBP1s-GFP expression was reduced in F/G-expressing syncytia (C). Also in line with the findings for Vero76 cells (see Fig. 4), XBP1 splicing was increased in F/G-positive A549 cells if cell-cell fusion was blocked by NH_4Cl (D).

Fig. S4

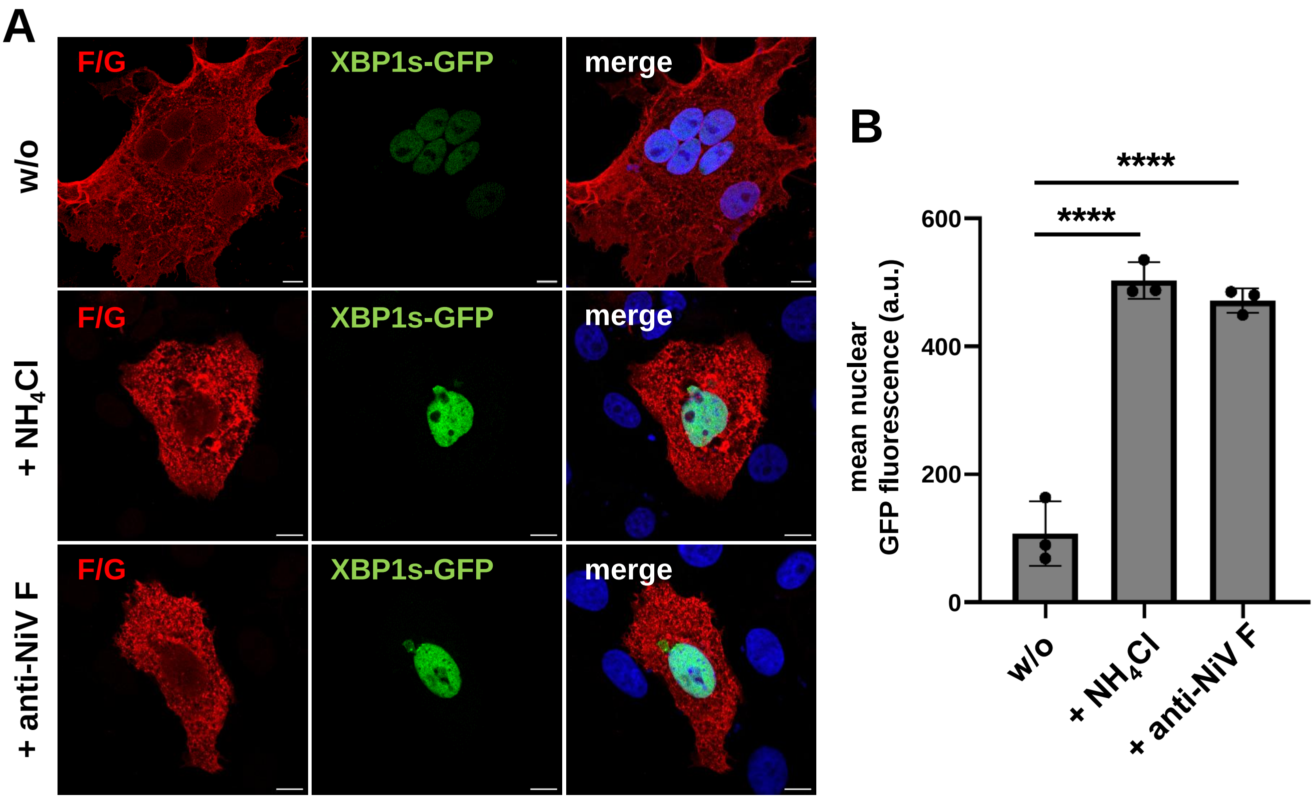

Figure S4. Effect on XBP1 splicing when syncytia formation is blocked by neutralizing antibodies. To rule out that the increased UPR activation in NH_4Cl-treated cells (see Fig. 4) was caused by an unspecific effect due to endosomal perturbation rather than by blocking cell-cell fusion, syncytia formation was inhibited by NiV-specific neutralizing antibodies. **(A)** Vero76 cells were transfected for 4 h with plasmids encoding the XBP1u-GFP reporter and HA-tagged F and G. Then, 20 mM NH_4Cl or neutralizing anti-NiV F antibodies raised against VLPs containing the NiV M and F proteins [2] were added to block syncytia formation. At 20 h p.t., cells were fixed and immunostained. Glycoproteins (red) were visualized with an HA-specific antibody. XBP1s-GFP (green) was detected by autofluorescence. Nuclei were counterstained with DAPI (blue). Scale bars: 10 µm. **(B)** Mean nuclear GFP fluorescence (XBP1 splicing) in the NiV glycoprotein expressing cell population was determined (arbitrary units, a.u.) and is shown as mean ± SD. Statistical significance was calculated using one-way ANOVA and Tukey´s post-hoc test. ****, p≤0.0001.

As in NH_4Cl-treated cells, F/G-mediated cell-cell fusion was blocked in the presence of anti-NiV F antibodies. If syncytia formation was prevented by either NH_4Cl or neutralizing antibodies, nuclear XBP1s-GFP expression was significantly increased strengthening the idea that syncytia formation limits UPR activation.

Fig. S5

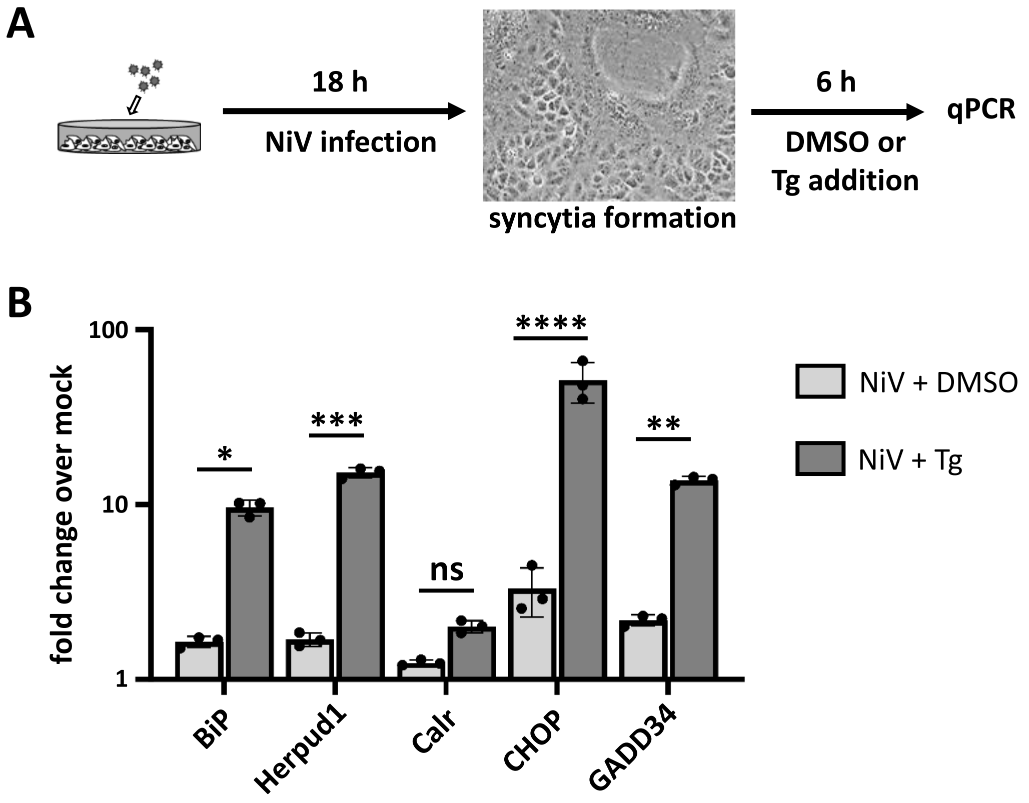

Figure S5. UPR induction in already formed NiV syncytia.
To rule out that cell-cell fusion prevents the onset of UPR, Vero76 cells were infected with NiV at an MOI of 0.1. At 18 h p. i., when syncytia had formed, Tg or DMSO was added and the infected cells were incubated for an additional 6 h before lysis and RNA extraction. Quantitative RT-PCR of UPR target genes was performed as described in the legend to Fig. 2. **(A)** Scheme of the experimental setting. **(B)** The fold change of DMSO and Tg-treated samples relative to mock-infected controls is shown ($2^{-\Delta\Delta ct}$). Data are represented as mean ± SD. Statistical differences between DMSO and Tg-stimulated samples were determined using a two-way ANOVA and Fisher´s LSD post-hoc test. *, p≤0.05; **, p≤0.01; ***, p≤0.001; ****, p≤0.0001, not significant (ns).

While UPR target genes were not upregulated in NiV-infected cells after 24 h (NiV + DMSO), Tg addition led to a similar induction of IRE1-, ATF6- or PERK-dependent target genes (NiV + Tg) as observed in non-infected cells (see Fig. 2A). The finding that exogenous UPR activation was not blocked in infected cells demonstrates that NiV-induced syncytia formation does not prevent the onset of the UPR. This further supports the idea that the lack of UPR induction by NiV is due to the fact that cell-cell fusion has a limiting or diluting effect on UPR inducers, such as the viral glycoproteins.

Fig. S6

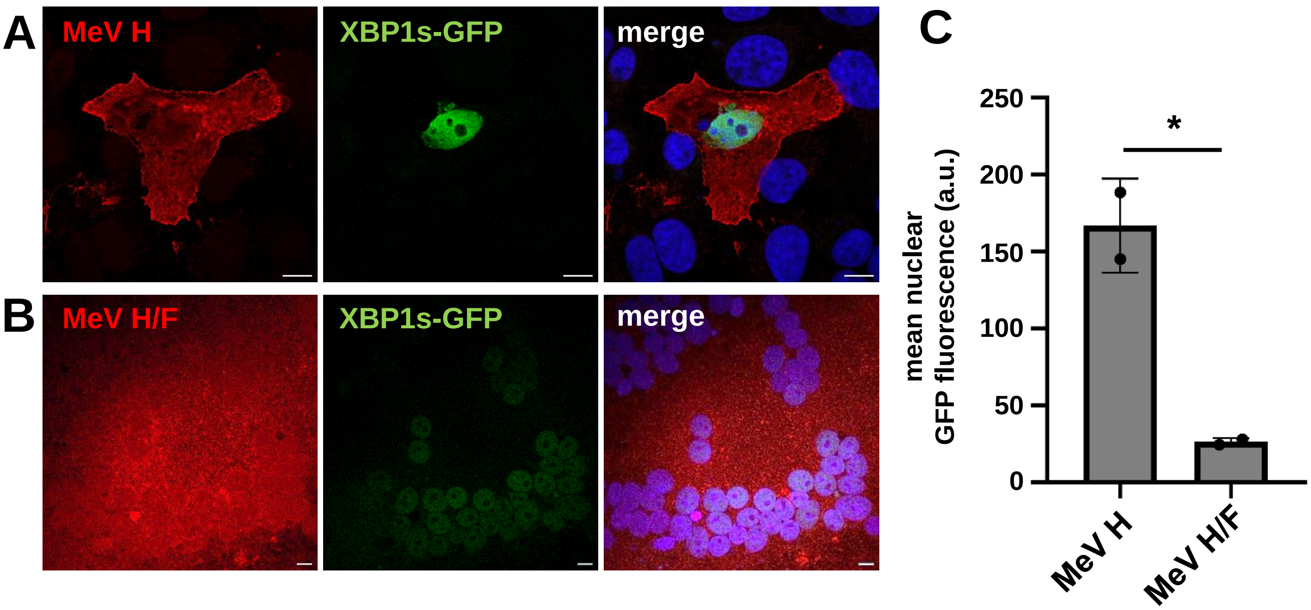

Figure S6. XBP1 splicing induced by the measles virus (MeV) glycoproteins.
(A, B) Vero76 cells were transfected with the XBP1u-GFP reporter plasmid and plasmids encoding the two MeV surface glycoproteins H and F, which we have described previously [3]. At 20 h p.t., cells expressing the MeV H protein alone (A), or syncytia formed by both MeV surface glycoproteins (MeV H/F) (B), were fixed with PFA and permeabilized with Triton X-100. MeV H (red) was detected with a monoclonal anti-H antibody (MAB8905, Chemicon). XBP1s-GFP (green) was detected by autofluorescence. Nuclei were counterstained with DAPI (blue). Scale bars: 10 µm. **(C)** Mean XBP1s-GFP fluorescence in the nuclear region of NiV glycoprotein positive single cells or syncytia is shown as mean ± SD of two independent experiments (>5000 cells per replicate). Statistical analysis was conducted using an unpaired t-test. *, $p \leq 0.05$.

Expression of the MeV H glycoprotein alone induced efficient XBP1 splicing. Upon coexpression of MeV H and F, large syncytia were formed and nuclear XBP1s-GFP expression was clearly reduced. These findings are in line with what was found in NiV G glycoprotein expressing single cells and NiV F/G-positive syncytia (see Fig. 3) and provide first evidence for the generalizability of our model that virus-induced cell-cell fusion can limit UPR activation.

Fig. S7

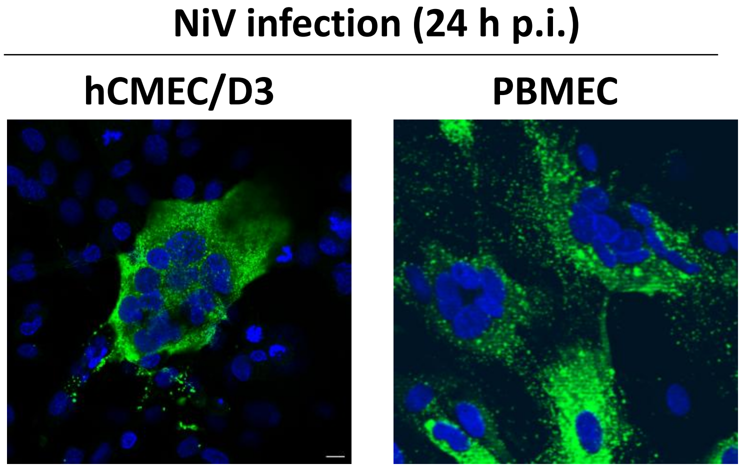

Figure S7. Syncytia formation in NiV-infected endothelial cells.
To monitor syncytia formation in human cerebral microvascular endothelial cells (hCMECs/D3) and primary porcine brain microvascular endothelial cells (PBMEC) isolated from pig brains [4], cells were infected with NiV at a MOI of 1. At 24 h p.i., cells were fixed and immunostained using polyclonal guinea pig anti-NiV antibodies and AF488-labelled secondary antibodies as described earlier [4]. Nuclei were counterstained with DAPI.

In line with the descriptions for *in-vivo* samples [reviewed in ref. 5] and confirming our earlier findings in primary endothelial cells [4, 6], NiV infection caused syncytia formation in both endothelial cell types. Although syncytia formation can vary greatly depending on the surrounding tissue, this supports the idea that cell-cell fusion can also occur in major target cell types of NiV infection *in vivo* and could thus influence activation of the UPR.
